# Immunotherapy Applications for Thymine Dimers and WT1 Antigen in Renal Cancers: A Comparative Statistical Analysis

**DOI:** 10.3390/jpm14060557

**Published:** 2024-05-23

**Authors:** Silviu Constantin Latcu, Razvan Bardan, Alin Adrian Cumpanas, Vlad Barbos, Flavia Baderca, Pusa Nela Gaje, Raluca Amalia Ceausu, Serban Comsa, Cristina-Stefania Dumitru, Raluca Dumache, Talida Georgiana Cut, Voichita Elena Lazureanu, Ligia Petrica

**Affiliations:** 1 Doctoral School, Victor Babes University of Medicine and Pharmacy Timisoara, E. Murgu Square, No. 2, 300041 Timisoara, Romania; silviu.latcu@umft.ro (S.C.L.); vlad.barbos@umft.ro (V.B.); 2 Department XV, Discipline of Urology, Victor Babes University of Medicine and Pharmacy Timisoara, E. Murgu Square, No. 2, 300041 Timisoara, Romania; cumpanas.alin@umft.ro; 3 Department II of Microscopic Morphology, Victor Babes University of Medicine and Pharmacy Timisoara, E. Murgu Square, No. 2, 300041 Timisoara, Romania; baderca.flavia@umft.ro (F.B.); gaje.nela@umft.ro (P.N.G.); ra.ceausu@umft.ro (R.A.C.); serban.comsa@umft.ro (S.C.); cristina-stefania.dumitru@umft.ro (C.-S.D.); 4 Angiogenesis Research Center, Victor Babes University of Medicine and Pharmacy Timisoara, E. Murgu Square, No. 2, 300041 Timisoara, Romania; 5 Department VIII, Discipline of Forensic Medicine, Bioethics, Deontology and Medical Law, Victor Babes University of Medicine and Pharmacy Timisoara, E. Murgu Square, No. 2, 300041 Timisoara, Romania; raluca.dumache@umft.ro; 6 Center for Ethics in Human Genetic Identifications, Victor Babes University of Medicine and Pharmacy Timisoara, E. Murgu Square, No. 2, 300041 Timisoara, Romania; talida.cut@umft.ro; 7 Department XIII, Discipline of Infectious Diseases, Victor Babes University of Medicine and Pharmacy Timisoara, E. Murgu Square, No. 2, 300041 Timisoara, Romania; lazureanu.voichita@umft.ro; 8 Department of Internal Medicine II, Division of Nephrology, Victor Babes University of Medicine and Pharmacy Timisoara, County Emergency Hospital Timisoara, E. Murgu Square, No. 2, 300041 Timisoara, Romania; petrica.ligia@umft.ro; 9 Centre for Molecular Research in Nephrology and Vascular Disease, Faculty of Medicine, Victor Babes University of Medicine and Pharmacy Timisoara, E. Murgu Square, No. 2, 300041 Timisoara, Romania

**Keywords:** renal cell carcinoma (RCC), proteomic kidney cancer biomarker, frameshift indels, immunotherapy, targeted therapy, immunohistochemistry (IHC), urological oncology, dark pyrimidine/cyclobutane pyrimidine/thymine dimers (PDs/CPDs/TDs), Wilms’ Tumor 1 (WT1) antigen

## Abstract

Renal cell carcinoma (RCC) remains incurable in advanced stages. Biomarkers have proven to be quite useful in cancer therapeutics. Herein, we provide a comparative/integrative statistical analysis of seminal immunohistochemistry (IHC) findings for Wilms’ Tumor 1 antigen (WT1) and thymine dimers (TDs), emerging as atypical, yet promising, potential biomarkers for RCCs. We assessed WT1/TD reactivity in adult RCC tumor cells, tumor microenvironment (TME), and tumor-adjacent healthy renal tissue (HRT). WT1 positivity was scarce and strictly nuclear in tumor cells, whereas TD-reactive tumor tissues were prevalent. We report statistically significant positive correlations between the density of reactive RCC cellularity and the intensity of nuclear staining for both biomarkers (WT1 − rho = 0.341, *p*-value = 0.036; TDs − rho = 0.379, *p*-value = 0.002). RCC stromal TME TD-positivity was much more frequent than WT1 reactivity, apparently proportional to that of the proper RCC cellularity and facilitated by extensive RCC inflammatory infiltration. TDs exhibited nuclear reactivity for most TME cell lines, while RCC TME WT1 expression was rare and inconsistent. In HRTs, TDs were entirely restricted to renal tubular cells, the likely cellular progenitor of most conventional RCC subtypes. In lieu of proper validation, these early findings have significant implications regarding the origins/biology of RCCs and may inform RCC therapeutics, both accounting for the high frequency of immunotherapy-permissive frameshift indels in RCCs, but also hinting at novel predictive clinical tools for WT1-targeted immunotherapy. Overall, the current study represents a meek yet hopefully significant step towards understanding the molecular biology and potential therapeutic targets of RCCs.

## 1. Introduction

Despite advances in molecular pathology and clinical management, kidney cancer remains one of the deadlier oncological diseases, particularly in advanced stages [1]. Renal cell carcinomas (RCCs) account for approximately 3% of cancers in adults worldwide [1]. Most RCCs are detected incidentally [2], with early detection resulting in improved survival rates due to prompt surgical intervention [3]. However, RCCs with advanced or recurrent presentations continue to show poor outcomes [4]. Moreover, overall mortality rates for kidney cancer have been steadily increasing by about 1% per year since the 1990s, despite progress in detection and treatment [3,4].

There have been significant strides in understanding RCCs at a molecular level, which are now known to represent a wide array of distinct tumor subtypes [5]. These vary in cellular origin, molecular drivers, and clinical behavior [6,7]. However, the complexity of these variations makes diagnosis and treatment increasingly complex. The World Health Organization's (WHO’s) 2022 Classification of Kidney Tumors now includes twenty-one distinct subtypes of malignant kidney tumors [5]. Yet, these refined classifications have not significantly improved patient outcomes, particularly for advanced RCCs, which remain incurable [8].

Immunohistochemistry (IHC) is a vital part of the routine pathological evaluation of kidney tumors [9,10,11,12], and RCCs in particular. Recent developments have provided a wide variety of potentially relevant RCC-associated proteomic targets [8,13,14,15]. However, herein there are substantial limitations, i.e., retrospective designs, limited samples, inconsistent clinical data, and an overall lack of standardization, with implicit extensive data heterogeneity [16]. As a result, no individual RCC biomarker or panel has been identified as clinically reproducible for progression prediction or response to systemic therapy [6,7,17,18,19]. Moreover, as a semi-quantitative immunoassay, IHC has conceptual limitations. It only offers isolated morphological frames, from within a much broader, dynamic system of molecular interactions, namely the RCC tumor microenvironment (TME) [20]. Despite nuanced characterizations of RCC tumor biology, current investigations have failed to demonstrate any added value for these biomarkers over existing prognostic models [21,22,23,24,25,26,27,28]. Nevertheless, IHC remains a useful clinical and experimental tool, facilitating the identification of novel research directions and offering crucial insights into RCC molecular biology [29,30,31,32,33]. Future therapeutics, guided by molecular pathway-driven targets, could offer impactful tools for advanced RCC treatment and clinical management.

Currently, there is an emphasis on further research in RCC molecular pathology due to the imperfect state of scientific knowledge in this field. Contemporary clinical management of RCCs is limited by inaccurate prognostic assessments, difficulty predicting RCC behavior and patient outcomes, limited treatment options, and low specificity of therapeutic interventions. Two uninvestigated nuclear markers, Wilms’ Tumor 1 antigen (WT1) [34] and thymine dimers (TDs) [35], have recently shown potential relevance in the RCC clinical context. WT1 may be a significant prognostic marker indicating mesenchymal dedifferentiation and increased tumor aggressiveness, while TDs, known for their role in cutaneous tumors, have demonstrated a novel expression pattern in RCCs. These markers could aid in developing more accurate prognostic assessments and personalized treatment strategies, improving the overall specificity of therapeutic interventions for RCC.

The Wilms’ Tumor 1 (WT1) antigen is an aggregate of the proteomic products that arise from its corresponding gene's transcription (~50 kilobases, 11p13) [36]. Initially, the WT1 gene was proposed as a specific proto-oncogene of metanephric nephroblastoma, i.e., Wilms’ tumor, the most frequent pediatric RCC subtype [36,37,38]. However, further research determined that while WT1 protein hyperexpression is evident in approximately 90% of nephroblastomas, only about 15% of these tumors present actual WT1 gene mutations [39]. Presently, the WT1 gene is recognized as remarkably complex, possessing at least 36 transcriptional proteic isoforms [40] and a multitude of cellular functions, some of which may be contradictory. These functions range from fetal organogenesis and maintaining metabolic homeostasis in various adult tissues [41,42,43], to roles in tumor suppression and mutational oncogenesis [44,45,46,47,48,49]. Despite these insights, the study of WT1 immunoreactivity remains scant in adult RCC patients, both in terms of immunoexpression patterns in RCC tumor tissues and in tumor-adjacent healthy renal tissues (HRTs) [34]. However, recent research [34] suggests that WT1 IHC could be potentially beneficial for differential diagnoses and prognostic predictions in adult RCCs. Moreover, WT1 demonstrates an intensely dynamic IHC expression pattern restricted to certain specific tissues in adults, yet maximal during urogenital embryogenesis, particularly during the ureteral bud invasion of the metanephric mesenchyme [40]. Thus, changes within these characteristic WT1 immunoexpression patterns within adult renal tissues may indicate a broader underlying process of cellular dedifferentiation, specifically epithelial-to-mesenchymal transition (EMT) [40]. With the recent development of novel WT1-targeted immunotherapy applications for RCC [34], there is an urgent need to carry out further WT1 investigations to more objectively predict individual responses to these therapies.

Thymine dimers (TDs) are genomic lesions caused by crosslinking between two adjacent thymine bases, specifically induced by ultraviolet (UV) radiation [50,51]. These lesions can cause specific UV mutations if left unrepaired [52,53,54], compromising genomic integrity and leading to dysfunction of various proto-oncogenes and tumor suppressor genes [55,56]—preferentially the TP53 gene [57,58,59]—and promoting further mutagenesis and oncogenesis [60]. Intriguingly, recent research has shown that “dark TDs” can continue to form for hours after initial UV irradiation, even in the complete absence of continued exposure, through distinct pathogenic mechanisms [61]. The biological significance of these dark TDs, however, has not yet been adequately evaluated [62]. Moreover, human fetal renal cells seem to be particularly ill-equipped enzymatically to manage the mutagenic effects of TDs [63]. Yet, there is currently little to no available data on the expression of TDs in HRT, RCCs, or any other non-cutaneous neoplasm [35]. These observations suggest the need for further investigations into the role and impact of these biomarkers in RCC and its management.

Our current paper deals with the topic of emerging unconventional proteomics in malignant renal tumors. Specifically, we aimed to further elaborate upon previous promising seminal IHC findings, focusing on the biological significance and comparative relationship between novel immunoexpression patterns for WT1 antigen [34] and TDs [35], in adult RCC tissues and their corresponding tumor-adjacent HRTs. To this end, we provide the current integrative retrospective statistical assessment.

## 2. Materials and Methods

### 2.1. Conceptual Design and Study Cohort

After obtaining the required ethical validations, we gathered and reexamined all relevant clinical data and available biological material (RCC paraffin-embedded tumor specimens), corresponding to a recent preexisting consecutive case series of 90 RCCs, treated surgically between 2016–2017, within the Arad County Hospital’s Urology Department. This entire cohort was comprised of neo-adjuvant treatment-naïve RCC patients who had directly undergone radical renal tumor excision, i.e., either partial or radical nephrectomy. Hospital records and all other available medical data were reassessed, from the pre-established individual databases, for WT1 antigen expression [34] and TDs, respectively [35], in order to document the overall initial clinical context of each patient, within our preliminary adult RCC cohort, i.e., patient sex, age at diagnosis and initial TNM staging.

After sampling and subsequent histological processing of the acquired RCC biological material, conventional microscopy was used to reassess the resulting HE-stained RCC sample slides. Targeted IHC staining case selection was achieved by individually assessing the entire initial RCC case cohort for study inclusion criteria conformity, i.e., the presence of both proper RCC tumor tissue, as well as tumor adjacent HRT, within each individual RCC sample slide. Herein, a total of only 30 RCCs met these pre-established inclusion criteria for further IHC staining. Moreover, an additional stratification of this final RCC study cohort was performed, based on the differential HRT expression of TDs, with a total of 12 TD-positive samples and 18 TD-negative samples.

### 2.2. Procedures and Definitions

Within this current IHC study, the biological material available for the initial cohort of consecutive RCC cases was limited to a single paraffin block of formalin-fixed RCC tissue section per case. We began processing these paraffin-embedded RCC specimens by firstly sampling the entire initial RCC cohort for further histological preparations. Using a standard microtome to slice the biological material, we obtained multiple 3 μm thick tumor tissue sections for each individual RCC case. Subsequently, these RCC tissue sections were transposed onto albumin pre-treated, silanized glass slides, then immersed in distilled water (one drop/slide) for repositioning and to prevent artifact formation. Once the excess liquid is removed, the slides require a 30-min thermal treatment at 58 °C, followed by an additional initial deparaffination stage (benzene, 58 °C, ≥30 min). Thereafter, the RCC tissue slides were stained with Hematoxylin-Eosin (HE), in an automated and well-standardized fashion, by using the Leica Autostainer XL system from Leica Biosystem Newcastle Ltd., Sheffield, UK. After HE staining, all of the resulting RCC tissue slides were reevaluated morphologically, using a Nikon E600 photon microscope (Boston, MA, USA) [34,35].

After preliminary IHC case selection, the final 30 RCC samples were then further categorized, based on their predominant tumor growth patterns, using the conventional morphology RCC subtyping system, i.e., 1—ccRCC, 2—pRCC and 3—chRCC, with 4—sarcomatoid dedifferentiation variants of RCC (svRCC) being reported separately [8]. We then assessed cellular/nuclear traits linked with aggressive RCC clinical behavior according to the WHO and International Society of Urological Pathology (ISUP) 2017 grading system G1–G4 [5]. These conventional morphological assessments of IHC-selected RCC samples were followed by similar targeted immunostaining protocols, performed in a standardized and entirely automated manner, using the Bond Max autostainer (Leica Biosystem, Newcastle Ltd., Sheffield, UK). Antigens were targeted using the following primary antibodies:

- WT1 proteins—ready-to-use, monoclonal, N-terminus targeted, clone WT49 from Leica Biosystem, Newcastle Ltd., UK. Requires predilution at room temperature (30 min). Incubation time: 20 min [34];
- Nuclear TDs—monoclonal anti-human TD mouse antibody, clone KTM53 from Kamiya Biomedical Company, Seattle, WA, USA. Dilution 1:10,000. Incubation time: 30 min [35].

Our IHC methodology was further validated by antigen-specific positive controls: internal for WT1, i.e., surrounding podocytes from within tumor adjacent HRT; vs. external for TDs, i.e., multiple in vivo, healthy human skin samples, untreated with additional UVR. After targeted IHC staining was finalized, RCC slide evaluation, quantification of quantitative and qualitative expression patterns, suggestive IHC image captures, and other relevant data collections were centralized. Cellular IHC positivity was defined as being exclusively nuclear for TDs, whereas both nuclear and/or cytoplasmic reactivity was deemed acceptable for WT1. Moreover, specific location (intratumoral vs. in tumor-adjacent HRT) and histological subtype of positive IHC cellularity, alongside several other targeted immunoreaction parameters were reported. For global intratumoral RCC staining, a simple “yes or no” IHC expression parameter was used, with nuance being provided by additional immunoreactivity scores, i.e., quantitative score (QS) vs. qualitative, intensity score (IS). Complete standardized score definitions are already available [34,35].

### 2.3. Statistical Analysis

IBM SPSS for Microsoft Windows (version 27.0), and Microsoft Excel were used to conduct the statistical analysis. The Kolmogorov–Smirnov test was used to assess the normality of the data. The mean value, which represents central tendency, and the standard deviation, which measures the dispersion of data, were used to describe normally distributed variables. Student’s *t*-test was used to examine the mean difference between the two comparison groups. The median and interquartile range (IQR) were used to characterize non-normally distributed data, presented in box plots, while the Mann-Whitney u-test was used to compare these variables. Considering the frequency assumption for the Chi-square test was not fulfilled, proportions were compared using Fisher's exact test. A correlation matrix was plotted to observe the association between variables of interest, while their statistical significance was represented by the Pearson or Spearman correlation coefficient “rho” and the associated *p*-value. Multivariate logistic regression was used to assess the impact of multiple factors on the presence of TDs in healthy tubular kidney tissue. A *p*-value below 0.05 was regarded as statistically significant.

## 3. Results

Overall, after preliminary histological processing of RCC tissue specimens, upon initial microscopic assessment, only 30 RCCs managed to meet the pre-established study inclusion criteria. Even though a total of 90 individual paraffin-embedded RCC samples were processed, the limited amount of available RCC biological material, i.e., one paraffin block/case, requiring repeated re-slicing, implies an intrinsically heterogeneous morphological environment among various slides for the same RCC sample. Furthermore, re-slicing the same limited RCC sample determined the loss of tumor-adjacent HRT on later slide iterations.

### 3.1. Microscopy and Immunoexpression Patterns

Morphological assessment of HE-stained RCC slides, provided a crude initial stratification of these 30 RCCs, i.e., using only the conventional RCC subtypes, with sarcomatoid variants reported separately. Thus, regarding predominant tumor growth patterns, we found 19 ccRCCs, 6 pRCCs, 3 chRCCs, and 2 svRCCs. Additionally, RCC nuclear grading showed 12 cases of G1, 12 cases of G2, 3 cases of G3, and 3 cases of G4.

**Figure 1:**
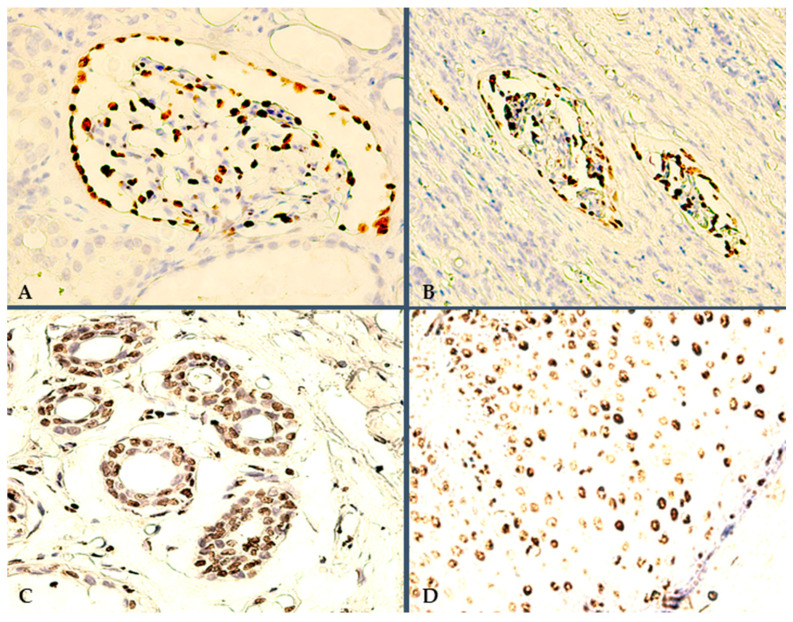
Positive controls for targeted IHC staining protocols: (**A**) 400×, WT1 internal positive control, i.e., reactive HRT podocytes; (**B**) 200×, WT1-positive podocytes in severely degenerated renal corpuscules, due to compression from the adjacent RCC tissue; (**C**) 400×, TD-positive dermal sweat glands and surrounding stromal endothelium; (**D**) 400×, TD epidermal immunoexpression pattern, i.e., intense nuclear TD positivity in keratinocytes.

Both our targeted immunoreactions were validated by their respective positive controls. Herein, for all 30 RCCs, the tumor-adjacent HRT consistently showed WT1 nuclear reactivity, specifically in podocytes and epithelial cells, i.e., within the parietal layer of Bowman’s capsule (Figure 1A). In fact, podocytes appear to maintain an intense WT1 expression, even among renal corpuscles undergoing evident degeneration (Figure 1B), i.e., the most resilient renal blood-filtration barrier cell line. Conversely, our TD-targeted IHC method was validated using an external positive control, i.e., multiple in vivo healthy skin samples. Representative nuclear TD reactions were seen in dermal sweat glands and stromal endothelium (Figure 1C), but most intensely in epithelial cells, with a peak intensity of final reaction products within the epidermis, particularly within the basal/para-basal cell layers (Figure 1D).

**Figure 2:**
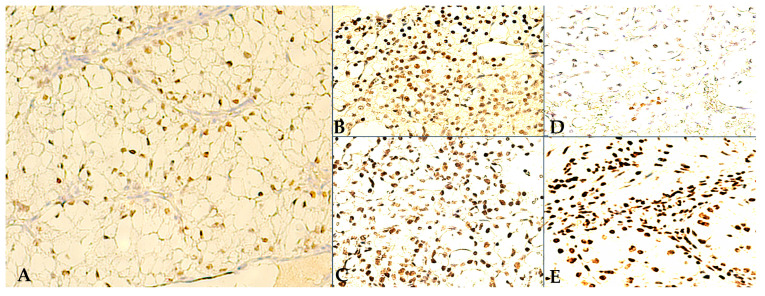
Intratumoral IHC staining: (**A**) 400×, WT1-positive ccRCC, moderate density (WT1-QS = 2), strong nuclear reactions (WT1-IS = 3); (**B**) Heterogeneous TD immunoreactivity pattern, with inconsistent RCC cell TD-reactivity in central tumor areas, yet with visible accumulation of density towards the tumor periphery, along the proliferation front (TD-QS = 3/IS = 2); (**C**) 400×, Homogenous TD immunoreactivity pattern, with diffuse, high density and strong intensity, TD-positive RCC tumor cellularity (TD-QS = 3/IS = 3); (**D**) 400×, Disorganized TD immunoreactivity pattern, with low density and weak intensity TD-positive RCC tumor cells, seen as sporadic nests within the TD-negative tumor stroma; (**E**) 400×, TD-positive RCC tumor cells and tumor stroma (pleomorphic cellularity).

Regarding intratumoral positivity, out of the 30 cases investigated, only 2 RCCs showed WT1 immunoexpression, whereas the broad majority, 23 RCCs, demonstrated TD reactive tumor tissues. Both WT1-positive RCCs were clear cell variants and showed moderate cell density (QS = 2), yet strong intensity of WT1 nuclear reactions (IS = 3), as seen in Figure 2A. However, clinically these RCC cases are quite different, i.e., a 62-year-old male with stage III, pT3aG2, ccRCC vs. a 58-year-old female with stage I, pT1aG1, ccRCC. Interestingly, the lower-grade RCC showed a more homogeneous WT1 staining pattern. Conversely, although both WT1-positive RCCs were also reactive to TDs, the higher-grade tumor showed a more significant TD expression pattern (QS = 3, IS = 2 vs. QS = 2, IS = 1).

**Figure 3:**
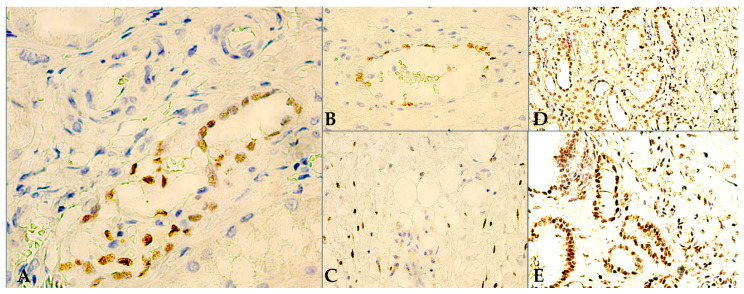
Tumor-adjacent HRT IHC staining: (**A**) 400×, WT1-positive tubular cellularity, low to moderate intensity nuclear staining; (**B**) 400×, stromal vein with mostly WT1-positive endothelial cellularity; (**C**) 400×, WT1-reactive stromal fusiform cells, morphologically reminiscent of fibroblasts; (**D**) 200×, high density of proximal and distal tubular cellularity, with weak/moderate intensity of nuclear TD expression, bordering TD-negative RCC tumor tissue; (**E**) 200×, various proximal/distal tubular structures, with moderate/strong intensity of nuclear TD immunoreactions, adjacent to non-reactive RCC cellularity.

In fact, amongst the 23 RCCs positive for TDs, microscopy revealed 3 main distribution patterns: (1) heterogeneous—TD-positive tumor cells are concentrated along the RCC proliferation front, and in the transitional areas, between tumor and HRT, in moderate/high density (QS = 2/3), and with moderate/high intensity of TD nuclear reactions (IS = 2/3), but are inconsistently expressed in the more central tumor areas (Figure 2B); (2) homogenous—diffuse, extremely dense (QS = 2/3), TD staining pattern, with inconsistent reaction intensity (IS = 2/3) (Figure 2C); (3) disorganized—a seemingly random, low density (QS = 1) and mostly moderate intensity (IS = 1/2), TD immuno-reactivity pattern (Figure 2D). Moreover, regarding intratumoral TD-positive RCC stromal cellularity, various lineages have shown nuclear TD-positivity, especially in homogenous TD-staining pattern RCCs, and/or in the presence of abundant inflammatory infiltrates on HE staining. In Figure 2E, we show TD-positive stromal/immune cells (i.e., fibroblasts, macrophages, lymphocytes) encircling TD-positive RCC cells. In contrast, the RCC intratumoral stroma was much less reactive to WT1, with only sporadic endothelial cells being positive at times.

Comparatively, when focusing solely on tumor-adjacent HRT expression patterns within the current IHC-stained RCC cohort, we report 6 WT1-reactive cases at this level, as well as 12 TD-reactive, respectively. For both targeted biomarkers, HRT expression was then further stratified based on specific cellularity involved, i.e., healthy kidney stromal expression (HSE) vs. healthy kidney tubular expression (HKTE), as follows: for WT1 HRTE, 4 cases had HSE in fibroblasts (Figure 3C) and endothelial cells (Figure 3B), and 2 cases had HKTE (Figure 3A); whereas for TD HRT expression, all 12 cases showed exclusively HKTE (Figure 3D,E). Furthermore, we encountered no glomerular TD-reactivity within the HRT samples evaluated.

### 3.2. Data Processing and Interpretation of IHC Results

In an effort to further integrate and better comprehend the previously reported IHC results, a statistical analysis of our emergent consolidated RCC database was performed, centered around the potential clinical significance of TD HRT expression patterns. The variables considered for analysis comprised: (1) background characteristics: age, age range, sex, cancer stage at diagnosis; (2) oncological characteristics: local extension (pT), positive lymph nodes (cN), distant metastases (cM), morphological RCC subtype and nuclear grade; (3) WT1 immunoexpression patterns: proportion of samples with intratumoral expression, WT1-QS for intratumoral expression (rare, moderate, or high density), WT1-IS intratumoral expression (weak, moderate, or strong), WT1 expression in healthy renal stroma; (4) TD immunoexpression patterns: rate of TD-positive intratumoral samples, TD-positive healthy stroma, TD-QS intratumoral expression, and TD-IS intratumoral intensity of expression (weak, moderate, or strong).

#### 3.2.1. Background Analysis

In Table 1, we review the background characteristics of our current RCC study cohort, stratified by the overall TD-positivity of tumor-adjacent HRT, i.e., HKTE for TDs. As previously reported, our study cohort consisted of 30 patients, with 12 (40%) exhibiting the presence of TDs in healthy kidney tubular tissues (HKTE+), as opposed to the remaining 18 RCC samples (60%), lacking TD-reactivity within the analyzed HRT samples (HKTE-). The mean age of RCC patients from within the HKTE-positive subgroup was 63.2 years, with a standard deviation of 7.5, whereas the mean age of TD-negative HKTE RCC patients was 67.2 years, with a standard deviation of 8.8. The difference in mean age between the two subgroups was not statistically significant (*p* = 0.207), while the distribution of sex between the two groups also showed no statistically significant difference (*p* = 0.215). In the TD HKTE(+) RCC subgroup, 50% were men and 50% were women, while in the HKTE(−) subgroup, 72.2% were men and 27.8% were women, without any significant differences between proportions. Regarding the RCC stage at initial diagnosis, there were no statistically significant differences between the TD HKTE(+) and HKTE(−) subgroups (*p* = 0.368). In the TD HKTE(+) subgroup, 41.7% were diagnosed at stage 1, 25% at stage 2, and 25% at stage 3. In the HKTE(−) group, 66.7% were diagnosed at stage 1, while 2 patients (11.1%) were at stage 2, and the remaining 22.2% were at stage 3.

#### 3.2.2. Clinical and IHC Findings

The oncological characteristics of the RCC study cohort, as reported in Table 2, seemingly corroborate the global conventional RCC subtype distribution, demonstrating a higher prevalence of ccRCCs in both groups, i.e., 58.3% of TD HKTE(+) RCCs and 66.7% of HKTE(−) RCCs respectively. The other RCC subtypes identified (pRCC, chRCC, and svRCC) associated varying percentages among the two subgroups, with pRCCs remaining the second most common RCC subtype within the TD HKTE(−) subgroup (27.8%). Regardless, there were no significant differences in RCC subtype stratification between the two groups (*p*-value = 0.149). The local extension (pT) of all individual renal tumors was also evaluated but with no significant differences between the HKTE(+) and HKTE(−) subgroups. The most prevalent degrees of local extension were pT3a (33.3%) for the positive group and pT1a (38.9%) for the HKTE(−) RCCs. Similarly, no relevant association was encountered between clinical staging parameters, i.e., the presence of positive lymph nodes (cN), with a *p*-value of 0.576, and distant metastases (cM), with a *p*-value of 0.212. These results suggest that there is no significant relationship between the presence of TDs in RCC adjacent HRT and local extension, lymph node involvement, or distant metastases. Regarding the nuclear grade, which is a measure of tumor cellular aggressiveness, the distribution was fairly similar between the two groups, i.e., 41.7% of RCCs from the TD HKTE(+) subgroup and 38.9% of TD HKTE(−) RCCs were labeled as G2 (*p*-value = 0.509).

In Table 3, we present the WT1 immunoexpression findings, seen within the RCC study cohort, as stratified by the presence of TDs in the healthy kidney tubular tissue of evaluated RCC samples. Intratumoral expression of WT1 was observed in 8.3% of HKTE(+) samples and 5.6% of HKTE(−) samples, with no significant difference between the two groups (*p*-value = 0.661). The density of WT1-positive intratumoral cellularity, as measured by the WT1-Quantitative Score (QS), also showed no significant difference between these subgroups (*p*-value = 0.908). Similarly, WT1 intratumoral nuclear reaction intensity, measured by the WT1-Intensity Score (IS), also showed no significant differences between the RCC groups (*p*-value = 0.453).

Regarding WT1 immunoexpression cellularity in tumor-adjacent HRT, WT1-positive endothelial cells were rarely noted, in only 16.7% of TD HKTE(+) RCC samples and in none of the TD HKTE(−) RCCs. Although there seems to be a difference in WT1 endothelial expression patterns among the groups, the *p*-value did not reach statistical significance (*p*-value = 0.072). WT1 expression in fibroblasts was also observed in 8.3% of HKTE(+) samples and 5.6% of HKTE(−) samples, with no significant difference between the groups (*p*-value = 0.765). Overall, WT1 expression in healthy renal stroma was found in 25.0% of HKTE(+) samples and 5.6% of HKTE(−) samples (*p*-value = 0.124).

**Figure 4:**
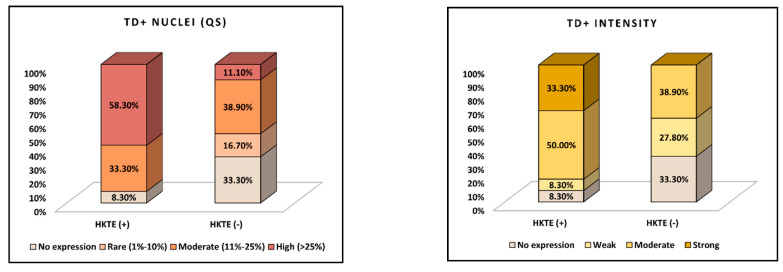
TD identification in the study RCC samples: quantitative and intensity scores.

In Table 4, we evaluate TD expression in the RCC study cohort, as stratified by TD HKTE(+) and HKTE(−) samples. The intratumoral TD immunoexpression variables analyzed include global TD-positivity, density of TD-positive RCC cellularity (TD-QS), and intensity of TD nuclear reactions (TD-IS). Global intratumoral TD-positivity was observed in 91.7% of TD HKTE(+) samples and 66.7% of HKTE(−) samples, yet this difference in positivity rates did not reach statistical significance (*p*-value = 0.112). Intratumoral TD-QS values showed a statistically significant difference between the TD HKTE RCC subgroups (*p*-value = 0.025). In the HKTE(+) group, 58.3% of samples had a high (>25%) cellular expression density for intratumoral TDs, while 33.3% had moderate density (11–25%), and 8.3% had no expression. In contrast, the HKTE(−) group had only 11.1% of samples with high-density intratumoral TD expression, 38.9% with moderate expression, 16.7% with rare (1–10%) TD-positive RCC cells, and 33.3% with no TD expression at all. Furthermore, the intratumoral intensity of TD nuclear reactions, as quantified by the TD-IS, also showed a significant difference between the RCC subgroups (*p*-value = 0.023). The TD HKTE(+) group had 33.3% of RCC samples with strong TD expression, 50.0% with moderate expression, 8.3% with weak expression, and 8.3% with no expression. In comparison, the HKTE(−) group had 0.0% of RCC samples with strong expression, 38.9% with moderate expression, 27.8% with weak expression, and 33.3% with no expression, as described in Figure 4.

#### 3.2.3. Statistical Analysis

**Figure 5:**
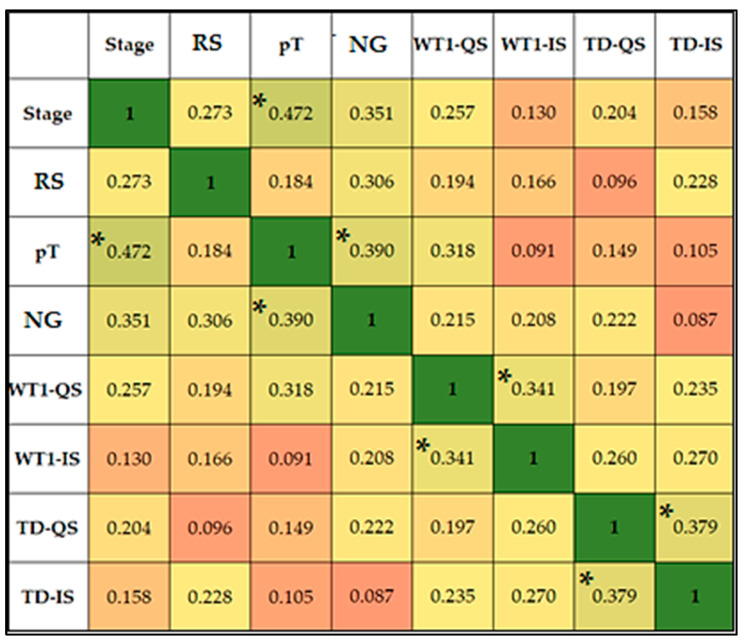
Correlation matrix. * Correlation is significant at the 0.05 level (two-tailed).

Figure 5 displays the Spearman correlation coefficients between various clinical parameters, including initial RCC stage and subtype (RS), pathological T stage (pT) and nuclear grade (NG), as well as intratumoral immunoexpression quantifiers: WT1 quantitative score (WT1-QS), WT1 intensity score (WT1-IS), TD quantitative score (TD-QS), and TD intensity score (TD-IS). We report statistically significant positive correlations between tumor stage and pT (rho = 0.472, *p*-value < 0.001), WT1-QS and WT1-IS (rho = 0.341, *p*-value = 0.036), TD-QS and TD-IS (rho = 0.379, *p*-value = 0.002), NG and tumor stage (rho = 0.351, *p*-value = 0.001), and between the NG and pT (rho = 0.390, *p*-value < 0.001).

The multivariate logistic regression analysis described in Table 5 assessed the impact of multiple factors on the presence of TDs in healthy tubular kidney tissue. Age, measured as a 1-year increase, showed no significant association with the presence of TDs (OR = 0.95, 95% CI = 0.86–1.04, *p*-value = 0.248). Similarly, the patients’ sex did not show a significant association (OR = 1.85, 95% CI = 0.52–6.53, *p*-value = 0.351). Initial stage and RCC subtype, using Stage 1 and ccRCC as reference categories, respectively, did not demonstrate significant associations with the presence of TDs in healthy tubular kidney tissue (*p*-values > 0.05). Nuclear grade, with a reference score of 1, showed no significant associations with the presence of TDs, nor did the WT1 immunoexpression parameters, i.e., intratumoral WT1 positivity, extratumoral WT1-positive endothelial cells, fibroblasts, and HRT stromal WT1 expression (*p*-values > 0.05).

Quantitative TD intratumoral expression, using no expression as a reference, showed a significant association for the high (>25%) density TD-expression group (OR = 3.62, 95% CI = 1.04–6.90, *p*-value = 0.040). The moderate (11–a25%) density group had a borderline significant association (OR = 3.17, 95% CI = 0.92–7.03, *p*-value = 0.061), while the low-density expression group did not show any significant association (*p*-value > 0.05). The intensity of intratumoral TD immunoreactions, using no expression as a reference, did not reveal significant associations for weak, moderate, or strong intensity groups (*p*-values > 0.05). Global intratumoral TD-positivity (Yes vs. No) had a borderline significant association with the presence of TDs in HRT (OR = 4.18, 95% CI = 0.96–8.42, *p*-value = 0.097). Thus, these results indicate that a high density of intratumoral TD expression is significantly associated with the presence of TDs in healthy tubular kidney tissue. Other factors such as age, sex, cancer stage, RCC subtype, nuclear grade, and WT1 immunoexpression parameters, did not show significant associations within this analysis.

## 4. Discussion

Our current paper, regarding the comparative and integrative analysis of IHC biomarkers (WT1 and TDs) expression patterns in adult RCC cellularity, TMEs, and tumor-adjacent HRT, is, to the best of our knowledge, the first of its kind. Corroborating previous findings, among the 30 adult RCC specimens currently evaluated, we found scarce intratumoral, strictly nuclear, WT1 positivity, i.e., in 6.66% (only two cases), using the N-terminus targeted, WT1 IHC antibody, clone WT49. Both WT1-positive RCCs were ccRCCs, with an identical pattern of WT1 intratumoral expression, i.e., moderate RCC positive cell density (QS = 2), but strong intensity of WT1 nuclear reactions (IS = 3), yet very difficult clinical presentations (62-year-old male, pT3aG2cN0M0 vs. 58-year-old female, pT1aG1cN0M0). Conversely, current results also support previous novel findings regarding the unexpected prevalence among RCCs of TDs, a specific kind of DNA pre-mutational lesion, which had previously only been reported in sun-damaged. Herein, the broad majority of our RCC cohort demonstrated TD reactive tumor tissues, i.e., 76.66% of cases (23 RCCs). Conversely, although both WT1-positive RCCs were also reactive to TDs, the higher-grade tumor showed a more significant TD expression pattern (QS = 3, IS = 2 vs. QS = 2, IS = 1). Furthermore, we report statistically significant positive correlations between the density of reactive cellularity and the intensity of nuclear immunoreactivity, for both biomarkers.

Cyclobutane pyrimidine dimers (CPDs), also known as TDs, are well-documented mutational events in melanoma and other skin malignancies, typically forming almost instantly when a UV photon excites a pyrimidine (cytosine or thymine) DNA base into a free radical state, which then chemically binds to another adjacent pyrimidine DNA base [50,64]. This phenomenon is perplexing in the case of RCCs and healthy kidneys, as they are not typically exposed to substantial sunlight. In fact, some evidence suggests that sunlight exposure may actually protect against kidney cancer development [65]. The traditional understanding of TDs as UV-dependent does not explain how these CPD/TD pre-mutational genomic lesions could occur in kidney cancer. A recent groundbreaking study has suggested a possible alternative route for CPD/TD formation outside of UV exposure, demonstrating the occurrence of CPDs/TDs in UVA/UVB-treated melanocytes hours after initial UV exposure has ceased [61]. Excited melanin molecules in the skin can stabilize free radicals created when exposed to UV radiation, generating reactive oxygen species (ROS) and nitrogen species. This "excited" melanin then causes a "late" mutation, termed a “dark” CPD/TD [61,62]. This might imply that a similar chemiexcitation-based, biochemical model could be at play in non-UV exposed epithelial tissues, like renal/urinary tissue, possibly involving a distinct renal/urinary tissue-specific protein as an energy vector and an alternative source for initial superoxide and nitric oxide production [35].

Despite the uncertain biochemical mechanism behind renal CPD formation, it has been shown in vitro that unrepaired UV-induced CPDs exhibit mutagenic effects in monkey renal cell cultures [66]. Furthermore, human fetal kidney cells are found to have a significantly diminished ability to repair CPDs via nucleotide excision repair (NER) compared to cells from various origins at the same developmental stage [63]. These findings collectively support the hypothesis that CPDs/TDs may play an active role in RCC carcinogenesis, potentially through tumor suppressor inactivation/proto-oncogene activation mutations or by exerting cytotoxic effects on immune response cells targeting the tumor [67]. The discovery of TDs in RCCs raises the possibility that free radicals or ROS might be involved in CPD/TD-driven mutations in kidney cancer. 

ROS have a complex role in cancer, participating in initiation, progression, and suppression, with their effects being highly context-dependent [68]. Smoking, a known source of ROS, is a well-established risk factor for primary RCC and progression to advanced RCC [69,70], while the intake of the antioxidant lycopene is associated with a reduced risk of kidney cancer [69,70,71]. It is possible that ROS from inflammation, infection, or environmental or dietary exposure could cause CPD/TD mutations [72]. Furthermore, molecules with antioxidant and inhibitory properties against TD formation, such as α-tocopherol (vitamin E) [73], isoflavone genistein [74], and resveratrol [75], may have potential applications in RCC prevention. Interestingly, a recent study using a murine model revealed IFN 1α/β to have a protective effect against UV-induced immunosuppression, and to enhance genomic photo-damage repair activity via NER gene induction [76]. Considering the current findings, TDs might have been overlooked as molecular therapeutic targets in metastatic RCCs treated with IFN 1α, by improving NER capabilities in TD-positive renal/RCC tissue.

Alternatively, CPD/TD pre-mutational lesions might be a downstream consequence of the unique genetics of RCCs. For instance, ccRCCs often have mutations or loss of the VHL gene, which typically results in elevated hypoxia-inducible factor 1α (HIF1α) accumulation, leading to significant changes in cellular metabolism including the suppression of oxidative phosphorylation and over-production of ROS [77,78]. These ROS can further stabilize HIF1α, damage mitochondria, and contribute to changes in tumor signaling. It is possible that these ROS may also contribute to mutagenesis and tumor evolution via the induction of CPDs/TDs and subsequent occurrence of TD-signature mutational events [77,79].

Similarly, WT1 gene expression is induced by hypoxia, with evidence showing that HIF1α directly transactivates WT1 transcription [80], suggesting a role for WT1 in the physiological response to ischemia [81]. After cardiac ischemia, WT1 expression is reactivated in the adult epicardium, potentially generating new coronary vasculature and cardiomyocytes [82]. In the oncological setting, WT1 expression has often been documented in the vasculature and stroma of various adult cancers [40]. In xenograft models of melanoma and lung cancer, WT1 expression was found in the host vasculature and stroma invading the tumor, and WT1 deletion led to impaired tumor growth and metastasis. Therefore, both of these unconventional biomarkers may hold significant potential for the development of impactful clinical applications in RCC.

Cancer, regardless of its origin, can be fundamentally defined by its ability to evade the immune system. Various molecular changes within specific cell lineages act as triggers for cancer development, often in relation to an imbalance in the TME. This imbalance typically lies between tumor-suppressing immune responses and proliferative inflammatory signals that promote cancer [20,83]. RCCs are known for their active TMEs, characterized by strong immune responses and extensive neo-angiogenesis. They exhibit a diverse range of cells, including stromal fibroblasts, immune-inflammatory infiltrates, arterial myocytes, and more. These cells continually interact with the emerging malignant RCC cells, creating a dynamic relationship that shapes the landscape of the RCC TME [35,84].

Current research efforts aim to understand the complexities of this TME heterogeneity in relation to RCC. Insights gained may shed light on the mechanisms involved in RCC's molecular biology [20]. Within this area of active research, we report intratumoral stromal TD-positivity as occurring preferentially in RCC specimens also demonstrating homogeneously dense and intensely immunoreactive TD-positive RCC tumor tissues, particularly when abundant intratumoral inflammatory infiltrates were present. Moreover, we found that most of the constitutive, biologically active, TME cell lines (i.e., fibroblasts, arterial parietal myocytes, and endothelial cells, as well as infiltrating immuno-inflammatory cells, such as lymphocytes and macrophages) exhibited nuclear TD immunoreactivity. Thus, it seems likely that these pre-mutagenic lesions could play an important, yet unknown, role in RCC carcinogenesis, proliferation, and dissemination. This may occur by disrupting the TME's anti-tumor immune responses via immune cell cytotoxicity [67,85]. Conversely, regarding WT1 expression, the RCC TME was found to be much less reactive, with only sporadic WT1 positive endothelial cells having been inconsistently encountered.

Furthermore, regarding tumor-adjacent HRT immunoreactivity, we found significant differences between the expression patterns of the two targeted biomarkers. Firstly, regarding the definition of HRT positivity, we only accepted stromal (HSE) and tubular (HKTE) immunoreactions for WT1. This is due to the fact that it has already been well-established that adult HRTs will characteristically and consistently manifest WT1 glomerular immunoreactivity, in podocyte populations. As expected, all 30 RCCs had positive internal controls, i.e., WT1 nuclear positivity in HRT podocytes, thus further validating our IHC method for WT1. Contrastingly, none of the HRT samples evaluated were capable of demonstrating glomerular TD-reactivity. Overall, we report 6 WT1-positive HRT cases, and 12 TD-reactive HRT specimens, respectively. Focusing on specific immunoreactive HRT cellularity subtypes encountered, we report that WT1 expression in HRT was both stromal (four cases), i.e., WT1 nuclear reactivity in fibroblasts and endothelium, as well as tubular (two cases); whereas for all 12 TD-reactive HRT cases, expression patterns were exclusively tubular. We must therefore highlight the fact that, remarkably, in all positive tumor adjacent HRTs, TDs were in fact entirely restricted to renal tubular cells, the likely cellular progenitor of most conventional RCC subtypes [72].

As for simultaneous positivity to both biomarkers in HRT samples, only four specimens were reported, i.e., all HKTE for TDs, with three HSE cases and one HKTE for WT1. The only case showing simultaneous WT1/TD HKTE was a 68-year-old female, with a stage 1 chRCC, pT2bG2cN0M0, negative for intratumoral WT1, but positive for TDs (QS = 3; IS = 2). For now, the interpretation of these novel, combined, W1/TD immunoexpression patterns in tubular HRT remains unclear. Similarly, even though the statistical analysis provided managed to indicate that a high density of intratumoral TD expression is significantly associated with the presence of TDs in HRTs, this conclusion is limited by the study's small cohort size. Even so, overall intratumoral TD-positivity was more prevalent among TD HKTE(+) vs. (−) RCCs (91.7% vs. 66.7%), whereas intratumoral TD expression had significantly higher density and stronger intensity in TD HKTE(+) samples. Comparatively, no statistically significant differences could be identified between TD HKTE(+) vs. (−) RCC subgroups, when stratified by overall intratumoral WT1 expression (8.3% vs. 5.6%), as well as WT1-QS and/or WT1-IS. However, high levels of WT1 protein expression have already been documented in some leukemias and almost all types of solid adult tumors. Although it is unclear whether WT1 overexpression is a causal contributor to the carcinogenic state or its consequence, WT1 is undeniably a promising tumor-associated antigen with potentially revolutionary applications, including evaluation of prognosis, detection of minimal residual disease/relapse, and immunotherapy, which are currently under investigation [86,87,88,89].

Immunotherapy is an emerging field in cancer treatment with the potential to target non-dividing cancer stem cells. For immunotherapy to be effective, a patient’s adaptive immune system must be vigilant, aware of, and actively trying to eradicate cancer. The immune repertoire should be “on-target”, either identifying a crucial neo-antigen from a key truncal mutation (e.g., BRAFV600 epitope in melanoma) or being broad enough to recognize tumor mutant neo-antigens from most, if not all, cancer clones. Immune checkpoint inhibitor therapy has been already validated in the adjuvant context, demonstrating benefits in some individuals with advanced RCCs. However, RCCs are known to have relatively few mutations overall and often even fewer truncal driver oncogene mutations, thus raising questions about the molecular pathology behind the perceived responsivity of RCC patients to immunotherapy [72]. 

Conversely, it has been previously demonstrated that RCCs have genomes enriched for frameshift indels, i.e., DNA mutations where a single DNA base pair is added or removed, scrambling the codon sequence downstream of the mutation into proteomic nonsense [90]. This may create an abundance of neo-epitopes, which are attractive targets for the adaptive immune system. [90,91]. Frameshift indels have been identified as successful targets for immunotherapy in numerous cancers [92,93,94]. The current IHC study, in corroboration with previous seminal work [35], reports the surprising prevalence of an underappreciated cause of mutations in RCC: CPDs, or simply TDs. These CPDs/TDs represent single-base-pair DNA pre-mutational lesions, traditionally considered to be characteristically caused by exposure to UV radiation. This event can lead to a variety of mutations, including substitution by deamination, or a frameshift indel may occur during DNA replication, potentially accounting for the comparatively large frequency of frameshift indels in melanomas [72]. Building upon our current work, further research to confirm and extend these still unvalidated findings, regarding the immunoexpression patterns of TDs in RCCs [35], may help us better understand the still occult genomic causes of RCCs, and clarify the highly disputed reasons behind the reported effectiveness of immunotherapy in advanced kidney cancer.

In contrast, much earlier, WT1 was initially implicated as a proto-oncogene for hematological malignancies, leading to the development of the first anti-WT1 immunotherapy applications in this context [95]. Indeed, there are quite a few inherent advantages in using WT1 as a target tumor antigen, namely: abundant expression in a wide variety of neoplasms, but with a very selective expression in mature physiological tissues and a significant difference in expression levels between healthy and neoplastic tissues [95]. Additionally, WT1 is highly immunogenic, as both peptide-based [96,97,98,99,100,101] and DNA-based [102] immunizations have been shown to be effective in inducing WT1-specific cytotoxic T lymphocytes, capable of more easily identifying and destroying WT1-positive tumor cells. Phase I/II clinical trials of WT1 peptide-based immunotherapy have reported frequent clinical responses and significant tumor regression in leukemia, myelodysplastic syndrome, lung, and breast cancers [100,101,102,103]. In 2007, the first report of WT1 peptide vaccination in advanced RCC showed suppressed tumor growth and stable disease in two out of three patients [95]. More recently, vaccination with WT1 peptide-loaded dendritic cells combined with targeted therapy or conventional chemotherapy demonstrated safety and feasibility in advanced RCC and bladder cancer patients [88]. In a patient-derived RCC xenograft tumor model, WT1-specific CTLs regenerated from induced pluripotent stem cells (iPSCs) showed therapeutic efficacy. The transfusion of these CTLs significantly suppressed the growth of RCC, providing a rationale for the clinical application of this strategy to treat solid tumors [89].

All in all, the documented immunoexpression patterns for WT1 and TDs in RCC tumor cells, TMS, and tumor-adjacent HRT generate numerous hypotheses about the origins and biology of RCCs and, potentially, may even inform kidney cancer therapeutics. However, these initial observations require additional external validation and mandate further exploration. Therefore, the derived hypotheses remain, for the time being, merely speculations and necessitate extensive confirmation and investigation. If validated, our findings regarding the immunoexpression of TDs in RCCs could help explain the high frequency of immunotherapy-permissive frameshift indels in RCCs. Conversely, deeper and more nuanced analyses of WT1 protein expression patterns may hold the key to developing more accurate clinical tools for WT1-tarted immunotherapy response prediction and/or case selection.

## 5. Study Limitations

The study design of the present investigation, i.e., a retrospective IHC assessment of comparative immunoexpression patterns for unconventional biomarkers in proper RCC tumor tissue and tumor-adjacent HRT, allows only speculative observations about the pathogenic significance and biological implications of WT1 transcripts and pre-mutagenic TDs in RCC carcinogenesis, progression and therapeutics. Therefore, our main study limitation is conceptual, as we employed IHC, which is only capable of providing an isolated, static, morphological portrayal of proteomic expression patterns within, the complex and dynamic molecular TME background of RCCs. In fact, IHC is also inherently plagued by further technical limitations and interpretative challenges. Sensitivity and specificity depend on various factors: the clone and detection technique employed, the size and quality of the specimen analyzed, and the renal tumor grade. Integration and standardization of quantitative staining parameters and marker expression pattern analysis in IHC reports are crucial [8].

In this study, we used a fully automated IHC staining technique and well-validated antibody clones. For TDs, we used the KTM53 clone, the oldest commercially available IHC antibody targeting pyrimidine dimers, which was designed to specifically target CPDs non-discriminately, without reacting with 6–4 pyrimidine-pyrimidone photoproducts (6–4PPs). The KTM53 clone has been employed in previous TD-targeted immunostaining investigations, more often focusing on human skin [104,105,106], but also, sporadically, in renal tissue, albeit after in situ hybridization with mRNA-targeted T-T dimerized synthetic oligonucleotides [107]. We aimed to provide a standardized and transferable protocol for reporting IHC results using quantification scores. However, we must acknowledge the internal validation issues posed by the protocols used for staining technique, result quantification, and interpretation. The possibility of non-specific binding of TD-targeted antibodies to unrelated, structurally similar, RCC antigens remains a significant investigational pitfall of IHC. Thus, our results may be distorted by false-positive TD-immunoreactions to some extent.

Conversely, the IHC methodology used for WT1 protein staining within the current investigation of adult RCCs must also be taken into account. Traditionally, WT1 cellular positivity using older, polyclonal, C-terminus-targeted WT1 antibodies was defined as being exclusively nuclear. Therefore, for a long time, any cytoplasmic immunoreactivity for WT1 has been generally excluded as artifactual staining, and thus constantly underreported [35]. Further on, following the development of more specific, novel, monoclonal, N-terminus-targeted WT1 IHC antibodies, this long-standing investigational paradigm has been significantly challenged by the recurrent WT1 staining patterns more recently reported, i.e., both individual and/or concomitant nuclear and/or cytoplasm WT1 cellular immunoreactivity [108,109,110,111,112,113,114]. In fact, in light of subsequent scientific developments, this emerging cellular heterogeneity of WT1 proteomic immunoreactivity has also been corroborated and explained by recent molecular evidence. Nowadays, WT1 transcripts are regarded as major regulators of genomic cellular processes, i.e., transcription/translation, manifesting metabolomics intracellular shuttling properties, thus migrating between the nucleus and cytoplasm [42,115]. Nevertheless, WT1 proteins are still mainly distributed in the cell nucleus, being first and foremost transcription factors, with a C-terminus (holding the characteristic four zinc finger motifs), involved in DNA/RNA binding, transcriptional regulation, self-association, and a proline/glutamine-rich N-terminus capable of RNA recognition [116,117,118]. 

Moreover, we must highlight the importance of considering both nuclear and cytoplasmic staining patterns when using different WT1 IHC antibodies in RCC research. Conversely, it is seemingly essential to use both types of WT1 antibodies (C- and N-terminus targeted clones) in IHC staining initiatives, in order to better comprehend and more fully account for this variability [39]. In the current IHC investigation, we used solely a recent N-terminus targeted WT1 antibody (clone WT49), albeit for the first time [34], to investigate WT1 proteomic expression patterns in adult RCCs. Simultaneously, in order to address the aforementioned IHC staining variability reported for WT1 protein, the current study design formally acknowledged WT1 cytoplasmic reactions within the definition of WT1 cellular positivity. Even so, confoundingly, we only found a very small percentage of WT1 reactive tumors, i.e., two exclusively nuclear WT1-positive adult RCCs. Notably, none of these WT1-positive RCC specimens had demonstrated any concomitant cytoplasmic staining, nor was cytoplasmic staining disregarded in any of the negative RCC cases. Thus, despite the limited study cohort, our current results contradict pre-existing data regarding the increased prevalence of IHC cytoplasmic reactions when using N-terminus-targeted WT1 antibodies. Herein, our meek positivity rate may be a consequence of using a single specific IHC clone, without also investigating C-terminus WT1 targets. 

Furthermore, our validation of the IHC methodology for TD staining is also somewhat questionable, mainly due to the fact that our immunostaining protocol was confirmed by an external reference. Control in vivo healthy skin samples were stained directly, without any additional standardized UV radiation treatment beforehand. Unsurprisingly, most of these untreated samples were predominantly negative for TDs, with only sporadic diffuse of TD nuclear reactivity being observed, in limited areas. Thus, although corroborated by previous external data, the cutaneous TD expression patterns reported herein, i.e., basal epidermal cells, sweat glands, and stromal endothelium were in fact encountered by chance, as a result of hazardous, pre-biopsy, in vivo, environmental UV exposures.

Finally, the novel results reported in this study, for each individual biomarker, have not yet been thoroughly validated by other commercially available targeted IHC antibody clones, i.e., the WT1 C-terminal region (amino acids 431–450) targeted H-1 clone (Santa Cruz Biotechnology, Inc., Dallas, TX, USA) and/or WT1 N-terminal region (amino acids 1–181) targeted ab89901 clone (Abcam, Cambridge, UK) vs. the TD-targeted H3 clone (GeneTEX) and/or ab10347 clone (Abcam); nor did we achieve confirmation of proteomic expressions through additional molecular assays such as enzyme-linked immunosorbent assay (ELISA), Southern blot, and/or quantitative real-time polymerase chain reaction (qRT-PCR). Similarly, although a statistical analysis is provided, due to the modest cohort of RCC samples, the statistically significant correlations found are still unreliable and require further validation. We present this early IHC report in hopes of expediting the external validation process.

## 6. Conclusions

In conclusion, scarce, strictly nuclear, WT1 positivity of tumor cells, among the adult RCC specimens analyzed, as opposed to the concomitant broad prevalence of TD-reactive tumor tissues, within the same study cohort. Similarly, RCC stromal TME TD-positivity was much more frequent than WT1 reactivity, apparently proportional to that of the proper RCC cellularity and facilitated by extensive RCC inflammatory infiltration. Herein, the majority of biologically active TME cell lines exhibited nuclear TD immunoreactivity. Contrastingly, RCC TME WT1 expression was rare and inconsistent, i.e., solely sporadic WT1 positive endothelial cells. In HRT cellularity, we report that WT1 expression was both stromal (fibroblasts and endothelium), as well as tubular, whereas for all TD-positive HRTs, TDs were in fact entirely restricted to renal tubular cells, the likely cellular progenitor of most conventional RCC subtypes. Statistically significant positive correlations between the density of reactive RCC cellularity and the intensity of nuclear immunostaining were found, for both biomarkers. Additionally, a high density of intratumoral TD expression was significantly associated with the presence of TDs in HRT. The significance of these novel IHC expression patterns in RCCs remains to be determined. Even so, these early findings reported within our current paper generate numerous speculative hypotheses about the origins and biology of RCCs. Moreover, the unconventional biomarkers analyzed may ultimately prove to be clinically impactful, by providing novel treatment targets and thus better informing RCC therapeutics. Clearly, further research is imperatively needed to confirm, extend, and further comprehend the biological implications of these novel IHC patterns in RCCs.

## Figures and Tables

**Table 1:** Background characteristics of the study cohort, stratified by the presence of TDs in the healthy kidney tubular tissue of evaluated RCC samples.

Variables	HKTE(+) (*n* = 12)	HKTE(−) (*n* = 18)	*p-*Value
Age, years (mean ± SD)	63.2 ± 7.5	67.2 ± 8.8	0.207
Age range	51–71	55–84	-
**Sex**			0.215
Men	6 (50.0%)	13 (72.2%)	
Women	6 (50.0%)	5 (27.8%)	
**Stage at diagnosis**			0.368
1	5 (41.7%)	12 (66.7%)	
2	3 (25.0%)	2 (11.1%)	
3	3 (25.0%)	4 (22.2%)	
4	1 (8.3%)	0 (0.0%)	

Data are reported as *n* (%) and compared using the Chi-square test or Fisher’s exact test unless specified differently; SD—Standard Deviation; HKTE—Healthy Kidney Tubular Expression of TDs.

**Table 2:** Oncological characteristics of the study cohort, stratified by the presence of TDs in the healthy kidney tubular tissue of evaluated RCC samples.

Variables	HKTE(+) (*n* = 12)	HKTE(−) (*n* = 18)	*p*-Value
**Cancer type**			0.149
ccRCC	7 (58.3%)	12 (66.7%)	
pRCC	1 (8.3%)	5 (27.8%)	
chRCC	2 (16.7%)	1 (5.6%)	
svRCC	2 (16.7%)	0 (0.0%)	
**Local extension (pT)**			0.632
1A	3 (25.0%)	7 (38.9%)	
1B	2 (16.7%)	3 (16.7%)	
2A	2 (16.7%)	3 (16.7%)	
2B	1 (8.3%)	3 (16.7%)	
3A	4 (33.3%)	2 (11.1%)	
**Positive lymph nodes (cN)**			0.576
Yes	3 (25.0%)	3 (16.7%)	
No	9 (75.0%)	15 (83.3%)	
**Distant metastasis (cM)**			0.212
Yes	1 (8.3%)	0 (0.0%)	
No	11 (91.7%)	18 (100%)	
**Nuclear grade**			0.509
G1	3 (25.0%)	9 (50.0%)	
G2	5 (41.7%)	7 (38.9%)	
G3	2 (16.7%)	1 (5.6%)	
G4	2 (16.7%)	1 (5.6%)	

Data reported as *n* (%) and compared using the Chi-square test or Fisher’s exact test unless specified differently; HKTE—Healthy Kidney Tubular Expression of TDs; ccRCC—Clear Cell Renal Cell Carcinoma; pRCC—Papillary Renal Cell Carcinoma; chRCC—Chromophobe Renal Cell Carcinoma; svRCC—Sarcomatoid Variant Renal Cell Carcinoma.

**Table 3:** IHC findings for WT1 in the study cohort, stratified by the presence of TDs in the healthy tubular kidney tissue of evaluated RCC samples.

Variables	HKTE(+) (*n* = 12)	HKTE(−) (*n* = 18)	*p-*Value
**Intratumoral WT1 expression**			0.661
Yes	1 (8.3%)	1 (5.6%)	
No	11 (91.7%)	17 (94.4%)	
**Density of intratumoral WT1 positive cellularity (WT1-QS)**			0.908
High (>25%)	0 (0%)	0 (0%)	
Rare/Moderate (1–25%)	1 (8.3%)	1 (5.6%)	
No expression	11 (91.7%)	17 (94.4%)	
**Intensity of intratumoral WT1 nuclear reactions (WT1-IS)**			0.453
Strong	1 (8.3%)	1 (5.6%)	
Weak/Moderate	0 (0.0%)	0 (0%)	
No expression	11 (91.7%)	17 (94.4%)	
**WT1 expression in tumor-adjacent HRT—endothelial cells**			0.072
Yes	2 (16.7%)	0 (0.0%)	
No	10 (83.3%)	18 (100%)	
**WT1 expression in tumor-adjacent HRT—fibroblasts**			0.765
Yes	1 (8.3%)	1 (5.6%)	
No	11 (91.7%)	17 (94.4%)	
**WT1 expression in tumor-adjacent HRT—healthy stroma**			0.124
Yes	3 (25.0%)	1 (5.6%)	
No	9 (75.0%)	17 (94.4%)	

Data are reported as *n* (%) and compared using the Chi-square test or Fisher’s exact test, unless specified differently; WT1—Wilms Tumor 1 gene; HKTE—Healthy Kidney Tubular Expression of TDs; QS—–Quantitative Score (by number of positive nuclei); IS—Intensity Score (by intensity of immunoreaction).

**Table 4:** IHC findings for TDs in the study cohort stratified by the presence of TDs in the healthy tubular kidney tissue of evaluated RCC samples.

Variables	HKTE(+) (*n* = 12)	HKTE(−) (*n* = 18)	*p-*Value
**Intratumoral TD expression**			0.112
Yes	11 (91.7%)	12 (66.7%)	
No	1 (8.3%)	6 (33.3%)	
**Density of intratumoral TD positive cellularity (TD-QS)**			0.025
High (>25%)	7 (58.3%)	2 (11.1%)	
Moderate (11–25%)	4 (33.3%)	7 (38.9%)	
Rare (1–10%)	0 (0.0%)	3 (16.7%)	
No expression	1 (8.3%)	6 (33.3%)	
**Intensity of intratumoral TD nuclear reactions (TD-IS)**			0.023
Strong	4 (33.3%)	0 (0.0%)	
Moderate	6 (50.0%)	7 (38.9%)	
Weak	1 (8.3%)	5 (27.8%)	
No expression	1 (8.3%)	6 (33.3%)	

Data are reported as *n* (%) and compared using the Chi-square test or Fisher’s exact test, unless specified differently; HKTE—Healthy Kidney Tissue Expression of TDs; TDs—Thymine Dimers; QS—Quantitative Score (by number of positive nuclei); IS—Intensity Score (by intensity of immunoreaction).

**Table 5:** Multivariate logistic regression table assessing the impact of multiple factors on the presence of TDs in healthy tubular kidney tissue.

Variables	Odds Ratio (OR)	95% CI	*p*-Value
Age (per 1-year increase)	0.95	0.86–1.04	0.248
Sex (female vs. male)	1.85	0.52–6.53	0.351
**Stage (reference: Stage 1)**			
Stage 2	0.95	0.38–8.07	0.650
Stage 3	1.44	0.52–4.28	0.736
Stage 4	4.02	0.90–20.15	0.413
**RCC subtype (reference: ccRCC)**			
pRCC	0.68	0.27–2.24	0.742
chRCC	0.45	0.52–1.75	0.290
svRCC	3.71	0.52–16.6	0.306
**Nuclear grade (reference: G1)**			
G2	0.90	0.33–2.68	0.880
G3	1.17	0.67–5.90	0.512
G4	2.56	0.81–6.06	0.268
**WT1 immunoexpression**			
Intratumoral WT1 positivity (Yes vs. No)	1.60	0.25–8.30	0.630
WT1 endothelial cell positivity (Yes vs. No)	2.09	0.77–10.91	0.302
WT1 fibroblast positivity (Yes vs. No)	1.13	0.82–3.07	0.759
WT1 healthy stroma positivity (Yes vs. No)	3.44	0.92–18.50	0.180
**Quantitative TD intratumoral expression (reference: no expression)**			
Rare (1–10%)	1.39	0.36–2.81	0.664
Moderate (11–25%)	3.17	0.92–7.03	0.061
High (>25%)	3.62	1.04–6.90	0.040
**Intensity of TD intratumoral expression (reference: no expression)**			
Weak	2.18	0.86–6.29	0.220
Moderate	1.94	0.62–6.55	0.391
Strong	4.33	0.92–11.61	0.114
Intratumoral TD positivity (Yes vs. No)	4.18	0.96–8.42	0.097

OR—Odds Ratio; CI—Confidence Interval.

## Data Availability

The original contributions presented in the study are included in the article, further inquiries can be directed to the corresponding author.
